# Prevalence of HIV infection and its impact on clinical outcomes among patients admitted to the general intensive care unit at Laquintinie Hospital, Douala, Cameroon

**DOI:** 10.11604/pamj.2025.52.64.48092

**Published:** 2025-10-09

**Authors:** Clotilde Njall Pouth, Grace Dalle, Martine Calixte Nida, Ferdinand Ndom Ntock, Willy Bilogui Adjessa, Calixte Ida Penda, Else Carole Eboumbou Moukoko

**Affiliations:** 1 Faculty of Medicine and Pharmaceutical Sciences, University of Douala, Douala, Cameroon

**Keywords:** HIV infection, intensive care unit, clinical outcomes, prevalence

## Abstract

**Introduction:**

updating epidemiological and clinical data on people living with HIV (PLHIV) is essential to optimize strategies for prevention, screening, and clinical management. This study aimed to determine the prevalence of HIV infection and assess its impact on clinical outcomes among patients admitted to the general intensive care unit (ICU) at Laquintinie Hospital in Douala (HLD).

**Methods:**

we conducted a cross-sectional descriptive study involving 179 patients admitted to the ICU of HLD. Sociodemographic, clinical, and biological data were collected using a structured questionnaire and analyzed using R software. Univariate and multivariable logistic regression analyses were performed to identify factors associated with mortality.

**Results:**

the prevalence of HIV-1 infection was 26.3% (n= 47). The mean age of patients was 41.23 ± 7.2 years (range: 25-89), with HIV-negative patients more frequently aged over 65 years: 22.0% (n= 29) versus 8.5% (n= 4) among HIV-positive patients (p= .002). Males accounted for 52.0% (n = 93) of the overall population, while females were predominant in the HIV-positive group: 61.7% (n= 29) versus 43.2% (n= 57) in HIV-negative patients (p= .029). Unemployment was the most common occupational status at 52.0% (n = 93), with no significant difference by HIV status (p = .70); secondary education level was also common (50.3%, n= 90). Clinically, coma was the main reason for ICU admission (72.1%, n = 129). Compared to HIV-negative patients, HIV-positive patients more frequently presented with severe dehydration (10.6%, n= 5 vs 3.0%, n= 4; p=.04), severe anemia (36.2%, n= 17 vs 19.7%, n= 26; p= .02), and sepsis (44.7%, n= 21 vs 16.7%, n= 22; p < .001); meningoencephalitis (48.9%, n = 23) and pneumonia (23.4%, n= 11) were also more common. Overall mortality was 29.1% (n= 52), nearly twice as high among HIV-positive patients (44.7%, n = 21) compared to HIV-negative patients (23.5%, n= 31). In multivariable logistic regression, three factors were independently associated with mortality: severe anemia (aOR = 7.90; 95% CI: 1.52-50.8; p = .019), sepsis (aOR = 5.53; 95% CI: 1.06-36.0; p= .040), and pneumonia (aOR= 5.53; 95% CI: 1.06-36.0; p= .040).

**Conclusion:**

our findings indicate that, among patients admitted to the ICU, HIV infection presence of severe anemia, sepsis, was associated with a more severe clinical profile and higher mortality, particularly in the presence of severe anemia, sepsis, or pneumonia.

## Introduction

Human immunodeficiency virus (HIV) infection remains a major public health concern: in 2021, approximately 38.4 million people were living with HIV worldwide, two-thirds of whom resided in sub-Saharan Africa [1]. Despite the expansion of access to antiretroviral therapy (ART), the region continues to face high levels of morbidity and mortality, largely due to late diagnosis, severe immunosuppression, and serious opportunistic infections [2]. In Cameroon, the national HIV prevalence is estimated at 3.6% [3], but up-to-date epidemiological and clinical data are lacking, making it difficult to adapt strategies for prevention, screening, and management. Admissions to intensive care units (ICUs) are a stark indicator of disease severity and are often associated with septicemia, shock states, severe anemia, and infectious comas occurring at advanced stages of the disease [4,5]. Bakewell N *et al*. highlighted that late diagnosis significantly increases the risk of ICU admission and associated mortality [6]. Moreover, opportunistic infections such as toxoplasmosis and meningoencephalitis remain major causes of multi-organ failure and death in these patients [7]. Yet, the clinical characteristics and outcomes of people living with HIV admitted to ICUs in Cameroon remain poorly documented. In this context, having up-to-date local data is essential to optimize screening protocols, adjust ICU resource allocation, and improve clinical outcomes. This study aimed to determine the prevalence of HIV infection and assess its impact on clinical outcomes among patients admitted to the general intensive care unit at Laquintinie Hospital in Douala (HLD).

## Methods

**Study design and setting:** we conducted a cross-sectional descriptive study in the general intensive care unit (ICU) of Laquintinie Hospital in Douala (HLD), a second-category hospital and accredited HIV treatment center (CTA) that provides care for more than 6,000 people living with HIV in Cameroon. Data collection took place over a four-month period, from July to October 2024.

**Study population:** the target population included all patients aged over 15 years admitted to the ICU during the study period. Patients (or their legal representatives) who consented to participate and agreed to HIV testing were included. Those who refused HIV testing or declined participation were excluded. No formal sample size calculation was performed, as we conducted an exhaustive recruitment of all eligible cases during the study period.

**Data collection:** data were collected using a structured questionnaire that captured sociodemographic, clinical, and biological variables. HIV screening followed the national algorithm, using rapid tests (Determine® and ImmunoComb®), with ELISA confirmation in cases of discordance. For HIV-positive patients, CD4 counts, chest X-rays, sputum analysis, cerebrospinal fluid (CSF) examination, or brain CT scans were requested depending on clinical presentation. The research team recorded patient data daily from admission until discharge or death.

**Definitions:** a patient was considered HIV-positive if both rapid tests were reactive or if the ELISA confirmation test was positive. Mortality was defined as death occurring during ICU hospitalization. Comorbidity categories (e.g. sepsis, severe anemia, pneumonia) were based on clinical or biological definitions provided by the Cameroonian Ministry of Health. Severe dehydration was defined as the presence of ≥ 2 signs of shock and a weight loss > 10%. Severe anemia was defined as hemoglobin < 8 g/dL.

**Statistical analysis:** data were entered in Excel and analyzed using R version 4.4.2. Continuous variables were expressed as means ± standard deviation, and categorical variables as frequencies (n, %). Bivariate comparisons used Pearson´s chi-square or Fisher´s exact test. Mortality was assessed using univariate logistic regression, and variables with P < .05 (or of recognized clinical relevance) were included in a stepwise descending multivariable logistic regression model. Results were presented as adjusted odds ratios (aOR) with 95% confidence intervals (CI), and statistical significance was set at P < .05.

**Ethical considerations:** the study protocol was approved by the Ethics Committee of HLD (Ref. 1713/AR/MSP/HDL/SCM/CR). Verbal informed consent was obtained from each patient or their legal representative. Data were anonymized using alphanumeric codes, and all unused biological samples were destroyed according to national guidelines. All HIV-positive patients received immediate psychological counseling and were referred to the hospital's CTA for appropriate care.

## Results

**Figure 1:**
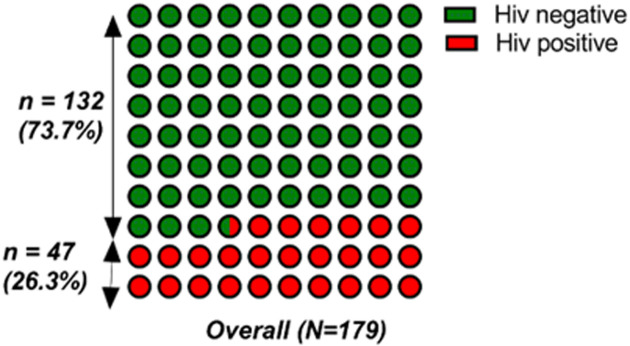
prevalence of HIV infection

**Prevalence of HIV infection:** the prevalence of HIV-1 infection was 26.3% (n = 47) (Figure 1).

**General characteristics of the study population:** the mean age of patients was 41.23 ± 7.2 years, ranging from 25 to 89 years. The majority of patients were over 65 years old (25.7%; n= 46), with 22.0% (n= 29) being HIV-negative and only 8.5% (n= 4) HIV-positive (p= .002). Males were more represented overall (52.0%; n= 93), with 56.8% (n= 75) among HIV-negative patients and 38.3% (n= 18) among HIV-positive patients (p= .029). Unemployment was the most common occupational status: 52.0% (n= 93), including 50.8% (n= 67) among HIV-negative and 55.3% (n= 26) among HIV-positive individuals (p= .70). A secondary education level was also predominant in 50.3% (n= 90) of the population, including 48.5% (n= 64) of HIV-negative and 55.3% (n= 26) of HIV-positive patients (p= .10). Most patients were married (53.1%; n= 95), with a higher proportion among HIV-negative (60.6%; n= 80) than among HIV-positive patients (31.9%; n= 15) (p= .003) (Table 1).

**Table 1:** sociodemographic factors of the study population

Factors	Total N (%)	HIV negative N(%)	HIV positive N(%)	P-value
**Age groups (years)**				002
< 25	12 (6.7%)	11 (8.3%)	1 (2.1%)	
[25-35]	33 (18.4%)	21 (15.9%)	12 (25.5%)	
[35-45]	30 (16.8%)	16 (12.1%)	14 (29.8%)	
[45-55]	29 (16.2%)	19 (14.4%)	10 (21.3%)	
[55-65]	29 (16.2%)	23 (17.4%)	6 (12.8%)	
> 65	46 (25.7%)	29 (61.7%)	4 (8.5%)	
**Gender**				029
Women	86 (48%)	57 (43.2%)	29 (61.7%)	
Men	93 (52%)	75 (41.9%)	18 (38.3%)	
**Profession**				70
Public sector	11 (6.1%)	8 (6.1%)	3 (6.4%)	
Private sector	61 (34.1%)	46 (34.8%)	15 (31.9%)	
Student	10 (5.6%)	7 (5.3%)	3 (6.4%)	
Unemployed	93 (52%)	67 (50.7%)	26 (55.3%)	
**Education level**				10
Primary	54 (30.2%)	39 (29.5%)	15 (31.9%)	
Secondary	90 (50.3%)	64 (48.5%)	26 (55.3%)	
Higher	27 (15.1%)	21 (15.9%)	6 (12.8%)	
Non-schooled	8 (4.5%)	8 (6.1%)	0	
**Marital status**				003
Single	58 (32.4%)	33 (25%)	25 (53.2%)	
Married	95 (53.1%)	80 (60.6%)	15 (31.9%)	
Divorced	1 (0.6%)	1 (0.8%)	0	
Widowed	25 (14%)	18 (13.7%)	7 (14.9%)	

The data are presented as counts (N), frequencies (n), and percentages (%). P-value: the Pearson chi-squared test was performed to assess the association between sociodemographic variables and the two groups of patients, with statistical significance set at p < 0.05.

Clinical profile of patients admitted to the intensive care unit (ICU) according to HIV status (HIV+ versus HIV-)

**Reasons for intensive care unit Admission According to HIV Status:** neurological emergencies, primarily coma (72.1%; n= 129), were the most frequent reasons for ICU admission, with no significant difference between groups. However, HIV-positive patients showed higher frequencies of severe dehydration (10.6% vs. 3.0%, p= .04) and severe anemia (36.2% vs. 19.7%, p= .02) among metabolic emergencies. Infectious emergencies revealed a significantly higher occurrence of sepsis among HIV-positive patients (44.7% vs. 16.7%, p < .001). Furthermore, hypovolemic shock was observed exclusively in HIV-negative patients (7.6%; n = 10) and in none of the HIV-positive patients (0%; n= 0) (p= .043) (Table 2).

**Table 2:** reasons for intensive care unit admission

Emergencies	Admission reasons	Total (N = 179)		HIV+ patients (N=47)		HIV- patients (N=132)		P-value
		N	%	N	%	N	%	
Neurological emergencies	Focal signs	38	21.2	6	12.8	32	24.2	20
	Convulsions	20	11.8	4	8.5	16	12.1	50
	Coma	129	72.1	30	63.8	99	75	14
Digestive emergencies	Acute febrile diarrhea	1	0.6	0	0	1	0.8	55
	Digestive hemorrhage	7	3.9	1	2.1	6	4.6	46
Metabolic emergencies	Severe dehydration	9	5	5	10.6	4	3	04
	Severe anemia	43	24	17	36.2	26	19.7	02
	Hyperglycemia	6	3.3	1	2.1	5	3.8	50
Respiratory emergencies	Respiratory distress	12	6.7	6	12.8	6	4.6	05
	Tracheobronchial congestion	1	0.6	1	2.1	0	0	26
Cardiovascular emergencies	Hypovolemic shock	10	5.6	0	0	10	7.6	04
	Collapse	1	0.6	0	0	1	0.8	73
	Hypertension	18	10.1	3	6.4	15	11.4	25
Infectious emergencies	SIRS	7	3.9	2	4.3	5	3.8	59
	Sepsis	43	24	21	44.7	22	16.7	< 001
	Septic shock	3	1.7	0	0	3	2.3	56

the data are presented as counts (N), frequencies (n), and percentages (%). P-value: the Pearson and Fisher chi-squared test was performed to assess the association between sociodemographic variables and the two groups of patients, with statistical significance set at p < 0.05; SIRS: systemic inflammatory response syndrome

**Clinical diagnoses associated with HIV infection in intensive care unit:** neuro-meningeal conditions were more frequent in HIV-negative patients, particularly stroke (37.12% vs. 12.77%, p= .002) and traumatic brain injury (25% vs. 10.64%, p= .039). In contrast, meningoencephalitis was more commonly observed in HIV-positive patients (48.94% vs. 9.09%, p < .001). Among digestive conditions, oral thrush was exclusively observed in HIV-positive patients (27.66% vs. 0%, p < .001). Regarding respiratory conditions, pneumonia was more frequent among HIV-positive patients (23.4% vs. 6.82%, p= .002) (Table 3).

**Table 3:** clinical diagnoses at intensive care unit admission

		HIV+ patients (N=47)		HIV-patients (N=132)		Total (N=179)			
	Clinical presentations		N	%	N	%	N	%	P-value
Neuro-meningeal conditions	Stroke		6	12.77	49	37.12	55	30.73	002
	Meningoencephalitis		23	48.94	12	9.09	35	19.55	< 001
	Head trauma		5	10.64	33	25	38	21.23	039
Digestive conditions	Oral thrush		13	27.66	0	0	13	7.26	< 001
	Hepatitis		0	0	1	0.76	1	0.56	75
	Others (Dysphagia)		1	2.13	0	0	1	0.56	50
Respiratory conditions	Pneumonia		11	23.4	9	6.82	20	11.17	002
	Pleuropneumonia		0	0	2	1.52	2	1.12	54
Dermatological conditions	Skin dermatosis		1	2.13	0	0	1	0.56	26
	Shingles		1	2.13	0	0	1	0.56	26
	Others (Prurigo)		1	2.13	0	0	1	0.56	6

The data are presented as counts (N), frequencies (n), and percentages (%). P-value: the Pearson and Fisher chi-squared test was performed to assess the association between sociodemographic variables and the two groups of patients, with statistical significance set at p < 0.05

**Clinical outcomes of patients in ICU according to HIV status:** among HIV-positive patients, 55.32% (n = 26) survived, compared to 76.52% (n= 101) among HIV-negative patients. Conversely, 44.68% (n= 21) of HIV-positive patients died, compared to 23.48% (n= 31) of HIV-negative patients (Table 4).

**Table 4:** clinical outcomes of patients

Evolution		HIV+ patients (n = 47)		HIV- patients (n = 132)		P-value
	N	N	%	N	%	
Living patients	127	26	55,32	101	76,52	0,006
Death patients	52	21	44,68	31	23,48	

The data are presented as counts (N), frequencies (n), and percentages (%). P-value: the Pearson chi-squared test was performed to assess the association between sociodemographic variables and the two groups of patients, with statistical significance set at p < 0.05

**Factors associated with mortality:** in univariate analysis, the factors significantly associated with increased risk of mortality were: convulsions, coma, hyperglycemia, severe anemia, respiratory distress, collapse, sepsis, septic shock, meningoencephalitis, traumatic brain injury, hepatitis, dysphagia, and pneumonia. After multivariable adjustment, only three factors remained independently associated with mortality: severe anemia (aOR: 7.90; 95% CI: 1.52-50.8; p= .019), sepsis (aOR: 5.53; 95% CI: 1.06-36.0; p= .04), and pneumonia (aOR: 5.53; 95% CI: 1.06-36.0; p= .04) (Table 5).

**Table 5:** univariate and multivariable logistic regression identifying factors associated with mortality

	Univariate logistic regression			Multivariable logistic regression		
Factors	COR	95% CI	P-value	AOR	95% CI	P-value
**Digestive emergencies**			87			90
Acute febrile diarrhea						
Digestive hemorrhage	1.09	0.38, 3.09	87	0.80	0.02, 20.6	90
**Neurological emergencies**			< 001			10
Focal signs						
Convulsions	7.77	2.70, 28.2	< 001	0.84	0.07, 9.69	88
Coma	3.08	1.47, 6.68	003	1.1	0.1, 8.4	4
**Metabolic emergencies**			< 001			046
Severe dehydration						
Hyperglycemia	8.63	4.00, 19.8	< 001	1.75	0.33, 8.92	50
Severe anemia	5.83	2.61, 13.7	< 001	7.90	1.52, 50.8	019
**Respiratory emergencies**			< 001			98
Tracheobronchial congestion						
Respiratory distress	11.4	4.99, 27.7	< 001	1.20	0.17, 9.02	85
**Cardiovascular emergencies**			055			72
Hypovolemic shock						
Collapse	2.51	1.18, 5.53	019	0.47	0.07, 2.98	42
Hypertension	1.78	0.77, 4.21	0.18	0.64	0.09, 4.69	66
**Infectious emergencies**			< 001			76
SIRS						
Sepsis	8.33	3.75, 20.2	< 001	5.53	1.06, 36.0	042*
Septic shock	2.90	1.40, 6.18	< 001	1.81	0.36, 9.47	47
**Neuro-meningeal conditions**			< 001			67
Stroke						
Meningoencephalitis	5.27	2.13, 14.5	< 001	0.58	0.07, 4.98	62
Head trauma	2.32	1.17, 4.64	< 001	1.61	0.31, 9.04	57
**Digestive conditions**			< 001			19
Oral thrush						
Hepatitis	9.51	4.27, 23.1	< 001	3.85	0.61, 27.4	16
Others (Dysphagia)	3.78	1.63, 9.16	002	0.58	0.11, 2.89	51
**Respiratory conditions**			< 001			04
Pleuropneumonia						
Pneumonia	8.33	3.75, 20.2	<0001	5.53	1.06, 36.0	04
**Dermatological conditions**			22			18
Skin dermatosis						
Shingles	0.67	0.34, 1.36	25	0.76	0.38, 1.56	5
Others (Prurigo)	1.15	0.64, 2.20	65	1.11	0.29, 5.46	9

cOR: unadjusted odds ratio; aOR: adjusted odds Ratio; CI: confidence interval

## Discussion

Patients living with HIV admitted to intensive care units (ICUs) often present with severe complications and high mortality rates, reflecting late diagnosis and limited access to care. In this context, it is essential to better understand the prevalence of HIV infection and assess its impact on clinical outcomes among ICU patients, particularly in countries like Cameroon, where HIV remains a major public health issue. By exploring the demographic, clinical, and biological characteristics of this vulnerable population, this study aimed to determine the prevalence of HIV infection and evaluate its clinical impact on patients admitted to the general ICU at Laquintinie Hospital in Douala (HLD). We found an HIV-1 prevalence of 26.3% (n= 47), with HIV-positive patients being younger, mostly female, and presenting a higher frequency of infectious comas and severe conditions, along with an overall mortality rate of 29.1%, rising to 44.7% among people living with HIV (PLHIV). In multivariable analysis, severe anemia (aOR = 7.90, 95% CI: 1.52-50.8, p= .019), sepsis (aOR= 5.53, 95% CI: 1.06-36.0, p= .040), and pneumonia (aOR = 5.53, 95% CI: 1.06-36.0, p=.040) were independently associated with mortality.

Our findings raise serious concerns. An HIV-1 seroprevalence of 26.3% was observed, with the majority (89.4%) unaware of their HIV status upon admission. This rate is significantly higher than that of the general population in Cameroon, estimated at 2.7% in 2018 [8]. Such a high rate may reflect the fact that ICU admissions often involve severe illnesses linked to undiagnosed immunosuppression due to HIV. The most affected age group was 25 to 45 years (72.2%), with a female predominance (54.4%). These results are consistent with a study conducted in Mali, which found a mean age of 40.9 years and a 67.6% female prevalence among HIV-positive ICU patients [9]. The over-representation of women may be explained by socio-economic and cultural factors, as well as biological vulnerabilities specific to women of reproductive age [10]. That 58.9% of the patients were single and 53.1% unemployed highlights the role of social determinants in vulnerability to HIV. A secondary level of education (55.6%) suggests that even basic education does not always protect against infection. Similar studies in sub-Saharan Africa have shown that socio-economic inequalities and limited access to information are key factors in the continued spread of HIV [11,12].

The fact that most patients were admitted at WHO clinical stage IV (62%) and with a CD4 count below 50 cells/mm^3^(69%) indicates advanced disease progression. This reflects delayed testing and limited access to antiretroviral treatment. A study from Côte d'Ivoire also reported that 82.4% of patients were severely immunocompromised at diagnosis [13], highlighting the need to reinforce early screening programs and community awareness initiatives [14]. The main causes of ICU admission-septicemia (22%), severe anemia (19%), and comas (16%)-reflect the severe complications of advanced immunodeficiency. Meningoencephalitis (51%) and toxoplasmosis (26.8%) were the most frequent diagnoses, in line with other African studies that identified these opportunistic infections as common in patients at advanced stages of HIV [15,16]. The observed mortality rate of 29.1% and mean hospital stay of 4.15 days underscore the severity of clinical presentations in this population. These figures are consistent with a study conducted in Senegal, where ICU mortality among HIV-positive patients reached 31% [17], and emphasize the urgent need to improve the management of HIV-positive ICU patients [18].

The presence of severe anemia, sepsis, and pneumonia formed a lethal triad determining prognosis in the ICU. In our multivariable model, severe anemia increased the risk of death nearly eightfold (aOR = 7.90; 95% CI: 1.52-50.8; p = .019), while sepsis and pneumonia each increased it more than fivefold (aOR = 5.53; 95% CI: 1.06-36.0; p= .040). These findings are consistent with those from a Brazilian cohort where sepsis was the leading prognostic factor for mortality among HIV-positive ICU patients [19]. They also align with a meta-analysis by Abioye *et al*. which showed that the severity of anemia proportionally increases all-cause mortality in PLHIV [20]. Moreover, the fatal impact of severe pulmonary conditions echoes recent data from French ICUs, where Pneumocystis jirovecii pneumonia remains associated with high hospital mortality despite therapeutic advances [21]. These converging findings highlight the need for early anemia detection, prompt sepsis management, and aggressive pneumonia treatment upon ICU admission to reduce what remains a largely preventable mortality. This study has certain limitations, including its monocentric design, which limits the generalizability of the findings to other hospitals or contexts. The short study duration and relatively small sample size may not capture seasonal variations or long-term trends, thus limiting a comprehensive assessment of the epidemiological and clinical profile of HIV-positive ICU patients.

## Conclusion

Our study highlights the high prevalence of HIV among patients admitted to the ICU at Laquintinie Hospital in Douala, particularly among undiagnosed young adults. The findings underscore the urgent need to improve early HIV testing and access to care for vulnerable populations. These data support the reinforcement of prevention, diagnostic, and management strategies for people living with HIV, especially in intensive care settings.

### What is known about this topic

- Mortality among HIV patients in the intensive care unit remains high despite antiretroviral therapy;
- Intensive care unit admissions often occur at advanced stages, with severe immunosuppression and opportunistic infections;
- Sepsis, pneumonia, and severe anemia are key prognostic determinants.

### What this study adds

- In Cameroon, 26.3% of intensive care unit admissions were HIV-positive, and 89% were unaware of their status;
- HIV-positive patients were mainly young women, with more frequent severe anemia, sepsis, and pneumonia than HIV-negative patients;
- Severe anemia, sepsis, and pneumonia independently increased hospital mortality (aORs: 7.9, 5.5, and 5.5, respectively).
